# Application of a Language Model Tool for COVID-19 Vaccine Adverse Event Monitoring Using Web and Social Media Content: Algorithm Development and Validation Study

**DOI:** 10.2196/53424

**Published:** 2024-12-20

**Authors:** Chathuri Daluwatte, Alena Khromava, Yuning Chen, Laurence Serradell, Anne-Laure Chabanon, Anthony Chan-Ou-Teung, Cliona Molony, Juhaeri Juhaeri

**Affiliations:** 1 Digital Data Sanofi Cambridge, MA United States; 2 Epidemiology and Benefit-Risk Department Sanofi Toronto, ON Canada; 3 Epidemiology and Benefit-Risk Department Sanofi Lyon France; 4 Global Pharmacovigilance Department Sanofi Lyon France; 5 Digital Data Sanofi Lyon France; 6 Epidemiology and Benefit-Risk Department Sanofi Bridgewater, NJ United States

**Keywords:** adverse event, COVID-19, detection, large language model, mass vaccination, natural language processing, pharmacovigilance, safety, social media, vaccine

## Abstract

**Background:**

Spontaneous pharmacovigilance reporting systems are the main data source for signal detection for vaccines. However, there is a large time lag between the occurrence of an adverse event (AE) and the availability for analysis. With global mass COVID-19 vaccination campaigns, social media, and web content, there is an opportunity for real-time, faster monitoring of AEs potentially related to COVID-19 vaccine use. Our work aims to detect AEs from social media to augment those from spontaneous reporting systems.

**Objective:**

This study aims to monitor AEs shared in social media and online support groups using medical context-aware natural language processing language models.

**Methods:**

We developed a language model–based web app to analyze social media, patient blogs, and forums (from 190 countries in 61 languages) around COVID-19 vaccine–related keywords. Following machine translation to English, lay language safety terms (ie, AEs) were observed using the PubmedBERT-based named-entity recognition model (precision=0.76 and recall=0.82) and mapped to Medical Dictionary for Regulatory Activities (MedDRA) terms using knowledge graphs (MedDRA terminology is an internationally used set of terms relating to medical conditions, medicines, and medical devices that are developed and registered under the auspices of the International Council for Harmonization of Technical Requirements for Pharmaceuticals for Human Use). Weekly and cumulative aggregated AE counts, proportions, and ratios were displayed via visual analytics, such as word clouds.

**Results:**

Most AEs were identified in 2021, with fewer in 2022. AEs observed using the web app were consistent with AEs communicated by health authorities shortly before or within the same period.

**Conclusions:**

Monitoring the web and social media provides opportunities to observe AEs that may be related to the use of COVID-19 vaccines. The presented analysis demonstrates the ability to use web content and social media as a data source that could contribute to the early observation of AEs and enhance postmarketing surveillance. It could help to adjust signal detection strategies and communication with external stakeholders, contributing to increased confidence in vaccine safety monitoring.

## Introduction

An adverse event (AE) is defined by the US Food and Drug Administration (FDA) as any undesirable experience associated with the use of a medical product (including vaccines) in a patient [1]. It can be challenging to assess uncommon or rare AEs in clinical trials due to the low number of patients enrolled in the trials and strict inclusion criteria. Therefore, postmarketing surveillance in a real-world setting is important to gain knowledge of any AE for manufacturers and regulatory bodies, including the FDA and European Medicines Agency (EMA), as well as international organizations such as the World Health Organization (WHO). New safety signals are defined by the EMA as “information on a new or known AE that may be caused by a medicine and requires further investigation” [2]. Organizations (pharmaceutical companies, drug regulators, distributors, wholesalers, and retailers) conduct postmarket pharmacovigilance surveillance both passively and actively, using systems including postauthorization safety studies, as well as voluntary and mandatory surveillance such as the US Centers for Disease Control and Prevention (CDC) or FDA Vaccine Adverse Event Reporting System (VAERS), MedWatch, Eudravigilance, and the WHO’s Vigibase. The reporting of suspected or observed AEs is mandatory for manufacturers and in many countries for health care professionals, however, the public may be unaware of AE reporting systems or feel a lack of obligation to report AEs, which in some cases may lead to delayed and incomplete records [3-6]. In addition, there can be a lag time between the reporting of an AE to the regulators and the information being available to the vaccine manufacturer from public sources. With the advent of the COVID-19 pandemic, with new vaccines being developed at speed and vaccines introduced in mass campaigns, assessing very rare AEs at the time of emergency use authorization has been difficult. Postmarketing AE reporting systems have become central to determining AEs and there have been some endeavors to decrease the lag time between reporting of an AE and information being available to the public.

Social media [7] has seen recent unprecedented growth in the numbers of users worldwide and in large populations of patients actively involved in sharing and posting health-related information [7]. This wealth of information has consequently led to data from such discussions being increasingly used for monitoring AEs [7-10], with the advantage of these occurring closer to real time than traditional postmarketing AE reporting systems. Many study groups have made use of natural language processing (NLP) and artificial intelligence methods [7,11,12], including the use of quantum computing [10,13], to observe AEs from social media data [7,10-12] and have had promising outcomes [10]. The COVID-19 pandemic has been at the heart of discussions on social media and the strong patient voice, discussing every concern surrounding COVID-19 vaccines in real time, has been documented in many studies [14-21]. Of note, Portelli et al [22] developed a tool that collected and analyzed public reaction to specific COVID-19 vaccines on 650,000 English language X (formerly Twitter; Twitter, Inc) posts (formerly tweets) since December 2020, including sentiment and AEs. Using a symptom extraction module, they showed news coverage had a high impact on topics discussed.

Safety surveillance will continue to evolve as there is currently a delay in reporting of AEs. A context-aware language model (LM), unlike a dictionary method, increases the sophistication of methods, for example, the interpretation of “corona” as a medical term and a nonmedical term [23]. Our work aims to detect AEs from social media to augment those from spontaneous reporting systems. The ability to monitor AEs on social media in real time has the potential to enhance postmarketing surveillance. Therefore, our objective was to monitor AEs and the related trends shared in social media and online support groups associated with COVID-19 vaccines using medical context-aware LMs.

The safety signals reported here have been denoted as AEs as they are mapped to Medical Dictionary for Regulatory Activities (MedDRA) preferred terms (PTs). However, they are not fully aligned with the definition of AEs that are subject to reporting to health authorities according to applicable regulations.

## Methods

### Overview

**Figure 1:**
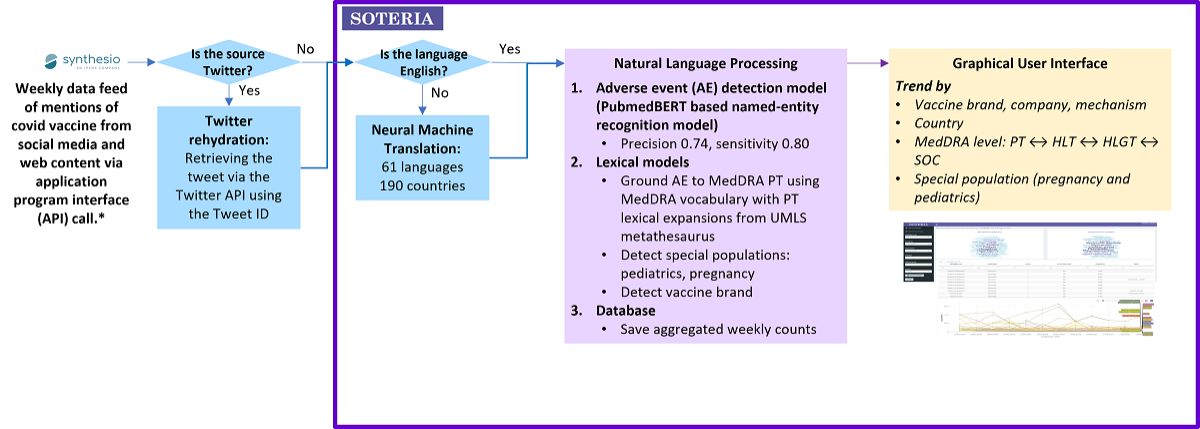
Soteria: language model powered web app to analyze web content related to COVID-19 vaccine AEs. AE: adverse event; API: application programming interface; HLGT: high-level group term; HLT: high-level term; MedDRA: Medical Dictionary for Regulatory Activities; mRNA: messenger ribonucleic acid; PT: preferred term; SOC: system organ class; UMLS: Unified Medical Language System. *Synthesio’s global partnerships guarantee that customers can complete their datasets with mentions from dozens of geo-specific social media and niche websites. Adhering to Twitter platform agreements and policies, Synthesio shares the ID, but not the tweets via the API. Using tweet IDs, tweets were recovered using the Twitter API, a process called “Twitter rehydration.”.

Our LM-powered web app is referred to as the Soteria web app from here onwards (Figure 1). Following machine translation to English, lay language AEs related to the safety of COVID-19 vaccines were detected using a named-entity recognition model and mapped to the International Council for Harmonization (ICH) MedDRA standards. Visual analytics as word clouds and line graphs were available on the graphical user interface to analyze across periods any trending of AEs (counts, proportions, and ratios) by COVID-19 vaccine brand, mechanism (messenger ribonucleic acid [mRNA], adenovirus vector, and protein), country, or special population (pediatrics or pregnant women). These can also be grouped by MedDRA hierarchy levels—system organ class (SOC), high-level group term (HLGT), high-level term (HLT), and PT, according to the latest version of MedDRA (23.0-24.0 in this analysis) as per Maintenance and Support Services Organization recommendations.

Contextual lexicons were generated to describe the pediatric population, pregnant population, and vaccine brand. Fuzzy matching detected if these topics were co-occurring with a medDRA PT mention.

### Soteria Web App

#### Neural Machine Translation

Non-English content in the data stream was translated to English before sending it to the LM for AE observation or detection. For each non-English sentence, the Amazon Translate neural machine translation service or Helsinki-NLP [24] translation models were used according to the source language (Multimedia Appendix 1) and the data source (data from X were translated using Helsinki-NLP, respecting X application programming interface [API] user agreement policies on not sending X posts to third parties). The translations from these machine translation models are continuously evaluated and validated by open-source communities [24] and Amazon Web Services. The Amazon Translate neural machine translation service is an off-the-shelf, usage-based service and Helsinki-NLP is noncommercial and open-source. Non-English sentences written using the standard English alphabet were removed due to known poor performance with machine translation models. COVID-19 vaccines monitored via the Soteria web app are listed in Multimedia Appendix 2.

#### AE Observation or Detection Model

We fine-tuned the pretrained LM, PubmedBERT [25], to perform a token-level classification task to obtain a named-entity recognition model using 2 publicly available datasets, adverse drug events (ADE)-Corpus-V2 [26] and psychiatric treatment adverse reactions (PsyTAR) [27]. The ADE-Corpus-V2 data contained 4271 sentences with AEs and 16,625 without, the PsyTAR data contained 4813 ADE mentions. The 2 datasets were combined and “machine labeled” for all ADE using the inside, outside, and beginning format and split into 70% training, 20% validation, and 10% test sets. The training was in 3 epochs with a batch size of 32 with a loss function of categorical cross-entropy. The final model’s performance was evaluated on the test set using precision (the proportion of sentences that had an AE among all sentences the algorithm had identified an AE), recall (the proportion of sentences the algorithm identified an AE among all sentences with an AE), and *F*_1_-score (harmonic mean of the precision and recall, which is a measure of accuracy).



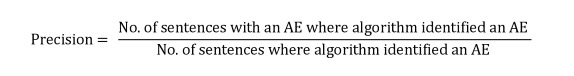





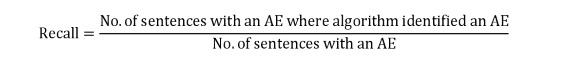





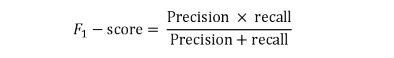



All analyses were performed in Python (version 3; Python Software Foundation) and with the module torch [28]. This named entity recognition model detected AEs in the Soteria web app (Figure 1).

The validation set was used to search for the hyperparameters that yielded the best performance for this set. The final model was trained on the entire dataset (training set plus evaluation set) and evaluated on a separate test set. Fivefold cross-validation was used for this process. After the final model had been trained, in the test phase, a new separate test set (not used during the training or validation process) provided an unbiased estimate of the model’s performance on unseen data. In order to account for the variability introduced by the random split, the model was trained and evaluated on each fold separately, with the results averaged across all folds to obtain a final estimate of the model’s performance [29].

#### MedDRA PT Lexical Expansion

We first performed a lexical expansion of MedDRA PTs using the Unified Medical Language System (UMLS) metathesaurus [30]. Each MedDRA PT was mapped with UMLS synonyms that have the same concept unique identifier but were from a different vocabulary other than MedDRA (Multimedia Appendix 3).

Lay language safety terms detected by the named-entity recognition model in the Soteria web app were mapped to MedDRA PT using these lexical expansions derived from the UMLS metathesaurus (Figure 1) using fuzzy matching. This mapping of AEs into MedDRA terminology allowed the harmonization of terms for a better understanding of patients’ chatting.

#### AE Trend Generation

Using mapped MedDRA terms, reports were generated in the Soteria web app as weekly or monthly AE count (for period and cumulative), proportion, ratio, and 95% CI around the ratio.



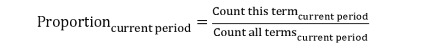





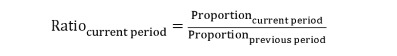





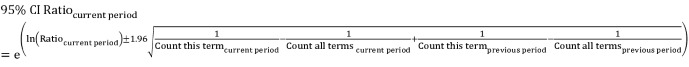



**Figure 2:**
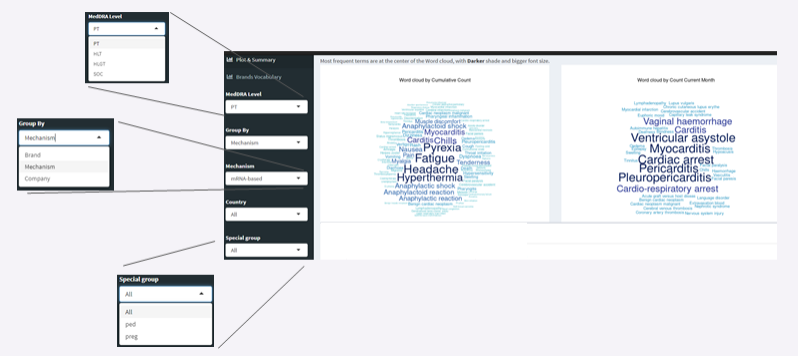
Detecting AEs for combinations of grouping by country, mechanism, brand names, and MedDRA levels (preferred term, high-level term, high-level group term, and system organ class). AE: adverse event; HLGT: high-level group term; HLT: high-level term; MedDRA: Medical Dictionary for Regulatory Activities; PT: preferred term; SOC: system organ class.

These metrics can be calculated for combinations of groupings by country, mechanism (mRNA, adenovirus vector, and protein), COVID-19 vaccine brand names, and MedDRA levels (PT, HLT, HLGT, and SOC; Figure 2).

**Figure 3:**
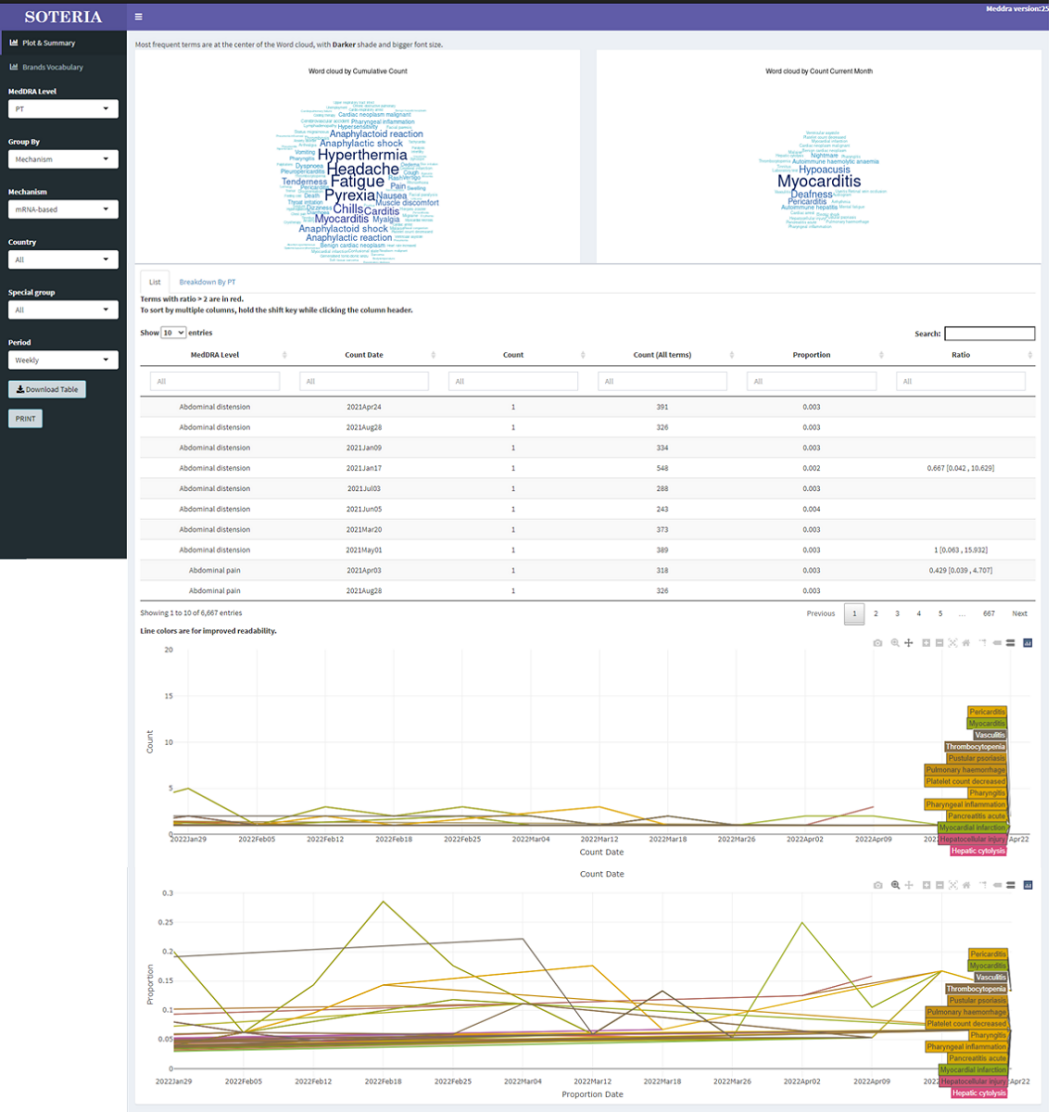
Soteria visual analytics. MedDRA: Medical Dictionary for Regulatory Activities; PT: preferred term.

These trends are presented as word clouds, tables with monthly top 50 AEs, and time trend line plots (Figure 3).

### Data Source

We obtained mentions of COVID-19 vaccine–related keywords from web content and social media via the API of the social listening tool from Synthesio Ltd using license-based data access via the API. Details of this tool can be obtained from Synthesio. Overall, mentions were collected from 190 countries in 61 different languages using a data query to identify mentions of a COVID-19 vaccine using COVID-19 vaccine–related keywords (Multimedia Appendix 4).

The social media types (as defined in the Soteria API) included are forums (excluding press releases), X, social networks, and comments and consumer opinions in the Soteria web app data stream via the Synthesio API. Adhering to X platform agreements and policies, Synthesio does not share the X posts via the API but only the X post ID. Using these X post IDs, we recovered the X post using the X API, a process at that time called “Twitter rehydration.” Synthesio Ltd does not share a list of social media platforms and websites. Considering the data volume, an analysis of the social media platform and website was not performed. While Synthesio Ltd API was used to collect social media and web content data for the Soteria web app, this data stream can be easily replaced by a similar provider’s API, X API, or a custom-built program to scrape web data (the authors do not recommend custom-built programs due to the nontrivial nature of the task in terms of technology, privacy, and compliance).

Data collection started on November 12, 2020, with automatic periodic weekly analysis of posts from the prior week and concatenation with all historic aggregated counts. In this analysis, results until April 2022 are presented (except for cumulative word counts, which are from October 2022).

### Ethical Considerations

The data analyzed does not contain any personal information but only the text data that mention a vaccine based on our query. The processing is an in-memory process that is completed within 24 hours and text data are not retained. Only aggregated counts of AEs were retained. This type of analysis does not require an institutional review board or ethics committee review. Sanofi follows the General Data Protection Regulation (GDPR) and Personal Information Protection Law (PIPL) and similar country-related policies for data protection. Synthesio also follows GDPR and other country-related policies for data protection and adheres to platform agreements or policies of all platforms they query from. For data and API acquired via X, Sanofi adhered to the X developer agreement and policy.

## Results

### AE Detection Model

The AE detection model had precision=0.76, recall=0.82, and *F*_1_-score=0.79 when evaluated on the test dataset (the AE Observation or Detection Model section provides details on the test dataset). This meant that 76% of the results were relevant and out of all positive predictions that could have been made, 82% were correct, resulting in 79% accuracy. Using the AE detection model, between November 2020 and December 2021, around 1 AE was observed at the MedDRA PT level for every 500 COVID-19 vaccine mentions and in 2022, this rate decreased to about 1 AE for every 2000 mentions. A considerably large portion of these AEs came from X data. Not every COVID-19 mention was associated with an AE.

### AE Trends Analyses

#### Number of AEs by Country

**Figure 4:**
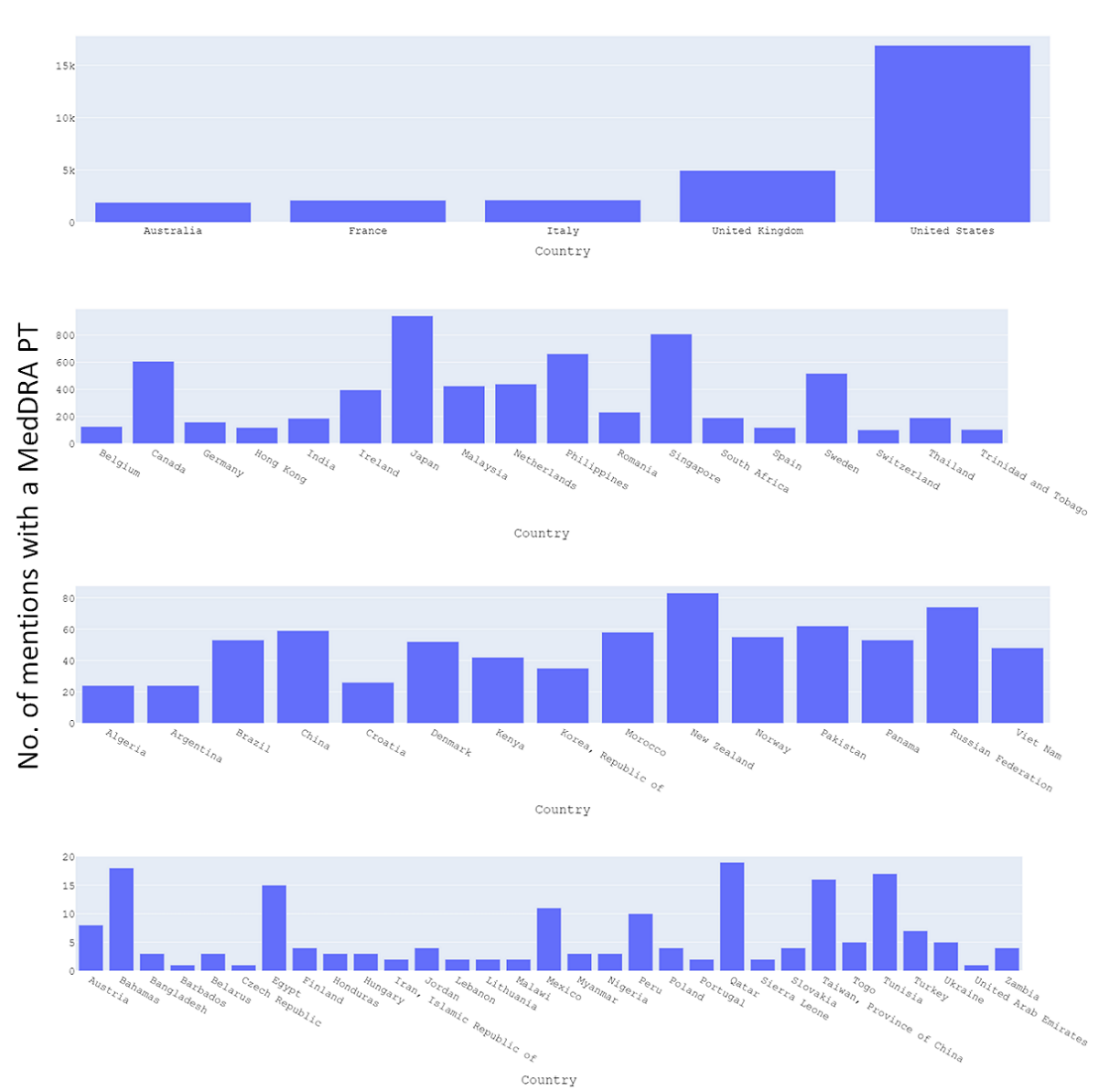
Distribution of number of mentions with an AE (detected as MedDRA PT) between November 2020 and April 2022 by country. AE: adverse event; MedDRA: Medical Dictionary for Regulatory Activities; PT: preferred term.

**Figure 5:**
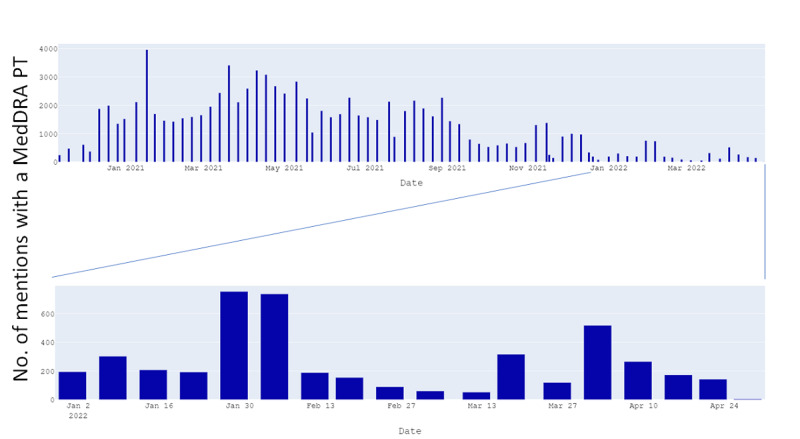
Distribution of number of mentions with an AE (detected as an MedDRA PT) between November 2020 and April 2022. AE: adverse event; MedDRA: Medical Dictionary for Regulatory Activities; PT: preferred term.

The countries with most AEs observed were the United States (>15,000), United Kingdom (~5000), Italy (~2000), France (~2000), Australia (~2000), Japan (~1000), Singapore (~800), Philippines (~650), and Canada (~600; Figure 4). The number of AEs observed varied each week and were less frequent in 2022 compared with 2021 (the distribution as of April 2022 is shown in Figure 5).

#### Number of AEs by Type of COVID-19 Vaccine (Platform and Brand)

**Figure 6:**
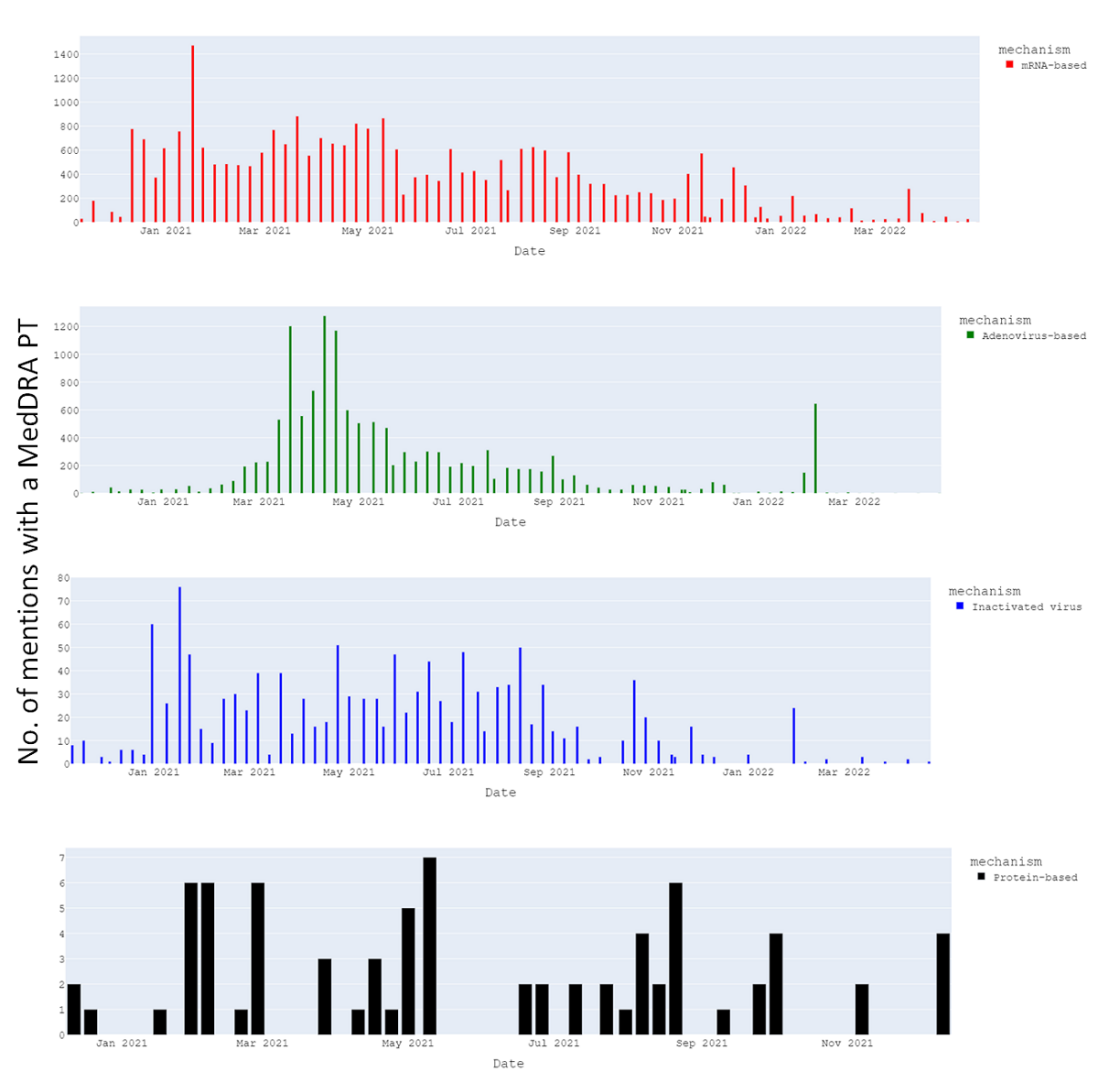
Distribution of number of mentions with an AE (detected as MedDRA PT) between November 2020 and April 2022 by the vaccine platform. AE: adverse event; MedDRA: Medical Dictionary for Regulatory Activities; mRNA: messenger ribonucleic acid; PT: preferred term.

The AEs observed were mostly related to mRNA vaccines and adenovirus vector vaccines while some AEs were related to inactivated virus vaccines and protein vaccines (Figure 6). AEs related to mRNA vaccines were first observed in December 2020 with a peak of 1400 in January 2021. AEs related to adenovirus vector vaccines increased from March 2020 with peaks of approximately 1200 in April and May 2021. AEs observed for mRNA vaccines stayed relatively high and stable during 2021, while in adenovirus vector vaccines’ decreased in the second quarter and over the year (Figure 6).

**Figure 7:**
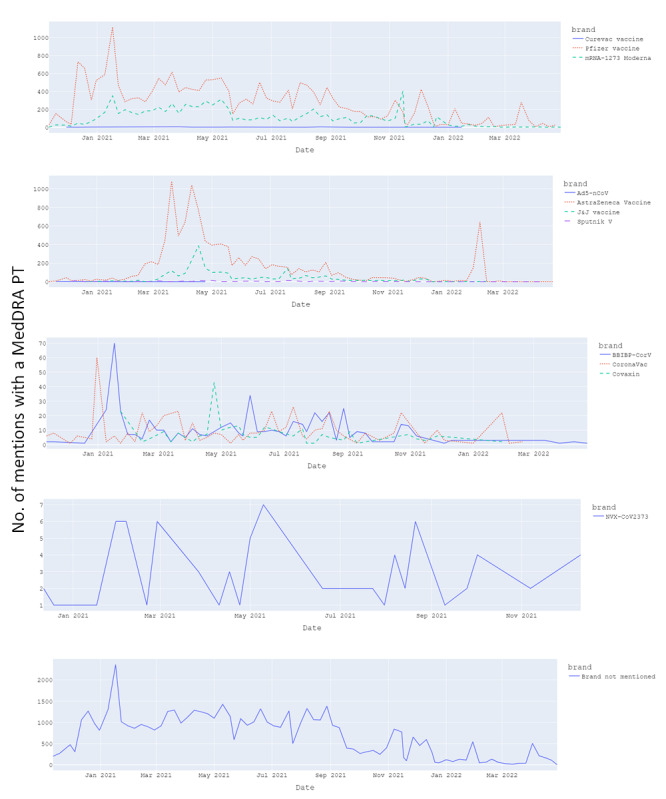
Distribution of number of mentions with an AE (detected as MedDRA PT between November 2020 and April 2022 by vaccine brand. AE: adverse event; J&J: Janssen (Johnson & Johnson); MedDRA: Medical Dictionary for Regulatory Activities; PT: preferred term; mRNA: messenger ribonucleic acid; NVX: Novovax.

Most AEs between November 2020 and April 2022 were associated with vaccines approved by the health authorities, that is, the mRNA vaccines from Pfizer-BioNTech and Moderna, and the adenovirus vector vaccines from AstraZeneca and Janssen (Johnson & Johnson). There were also many AEs where the administered vaccine names or pharmaceutical companies were not mentioned (Figure 7).

### Types of AEs

#### COVID-19 Vaccine Platform

The most frequently observed AEs for mRNA vaccines were headache, fatigue, pyrexia or hyperthermia, chills, nausea, pain or tenderness, and myalgia or muscle discomfort. The next most frequently observed AEs were those identified in the postmarketing setting—myocarditis and anaphylactic reactions (Multimedia Appendix 5).

The most commonly observed AE for adenovirus vector vaccines was thrombosis, which was also the AE identified in the postmarketing setting. The number of headaches and pyrexia, hyperthermia, or body temperature AEs all increased over time. Thrombocytopenia or platelet count AEs were associated with a specific syndrome “thrombosis with thrombocytopenia” and decreased over time (Multimedia Appendix 5).

#### COVID-19 Vaccine Brand

The AE “thrombosis” associated with the AstraZeneca vaccine (adenovirus vector) started to emerge in March 2021, and the word “COVID-19” related to the Sinopharm vaccine (inactivated virus) started to emerge in June 2021 (Multimedia Appendix 6).

The AEs observed associated with the Pfizer-BioNTech and Moderna vaccines (both mRNA) were similar, but a higher number of patients mentioned anaphylactic reactions regarding the Pfizer-BioNTech vaccine than the Moderna vaccine (Multimedia Appendix 7).

The AEs observed associated with AstraZeneca and Janssen vaccines (both adenovirus vectors) were different, with thrombosis being the most reported AE for the AstraZeneca vaccine and hyperthermia or pyrexia, fatigue, and headache followed by thrombosis and chills for the Janssen vaccine (Multimedia Appendix 7).

**Figure 8:**
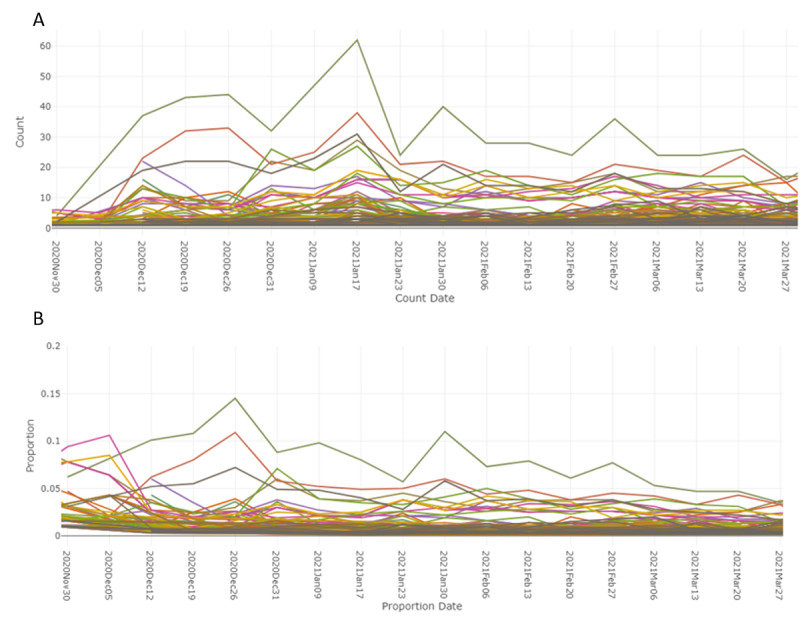
AEs from social media of mRNA COVID-19 vaccines from the time of launch in December 2020. (A) Count and (B) proportion. AE: adverse event; mRNA: messenger ribonucleic acid.

Anaphylactic reaction AEs started to trend in mid-December 2020 (under HLT “Anaphylactic and anaphylactoid responses”) shortly after the introduction of the mRNA vaccines (Figure 8).

Thrombo-embolic AEs started to trend on March 13, 2021, for adenovirus vector COVID-19 vaccines with HLTs including pulmonary thrombotic and embolic conditions, nonsite-specific embolism and thrombosis.

Myocarditis or pericarditis AEs started to trend for mRNA COVID-19 vaccines on May 1, 2021, with an increased ratio (proportion of noninfectious myocarditis HLT of the current period as compared to the previous one) on May 29, 2021. The proportion of myocarditis (count of myocarditis HLT divided by the count of all HLTs within the period) was the highest from September 2021 onwards (Multimedia Appendix 5).

### Subgroup Analyses

#### Pediatric Population

The most frequently observed AEs as of April 2022 for the pediatric population were carditis, myocarditis, hyperthermia, fatigue, and headache, followed by chills, anaphylaxis, and tenderness (Multimedia Appendix 8).

#### Pregnant Women

The most frequently observed AEs as of April 2022 for the pregnant women population were hyperthermia, fatigue, pyrexia, and headache, followed by chills, nausea, tenderness, and spontaneous abortion (Multimedia Appendix 8).

## Discussion

### Principal Findings

Monitoring the web and social media provides opportunities to observe both AEs and patient concerns around the safety of COVID-19 vaccines. We developed an LM powered web app (Soteria) to analyze web content related to COVID-19 vaccine AEs.

Using the Soteria web app, we were able to observe AEs associated with COVID-19 vaccines by country, vaccine brand, and by MedDRA level (PT, HLT, HLGT, and SOC) using data from social media and web content. Because social media and web content data are readily available and can be accessed and analyzed quickly, the Soteria web app could observe AEs in real time, much faster than detection using traditional spontaneous reporting systems, which have a larger time gap between the occurrence of an AE and the availability of data for analysis.

Results from our analyses were, in general, consistent with those from other sources. For example, our study showed that the number of AEs observed for COVID-19 mRNA vaccines remained relatively high and stable during 2021, aligning with the first COVID-19 vaccination campaigns, while for COVID-19 adenovirus vector vaccines the number of AEs decreased over the year, again consistent with the decreasing use of these types of vaccines. Widely known AEs for specific COVID-19 vaccines were also consistent with our findings using the Soteria web app, such as the thrombosis related to the AstraZeneca vaccine that started to emerge from March 2021 [31]. Other AEs observed using the Soteria app (anaphylactic reaction, myocarditis, and thrombosis) were also consistent with those communicated by different Health Authorities shortly before or concurrently [32-34]. Over time, there were fewer mentions of AEs observed in social media, reflecting the reduction of COVID-19 mass vaccination campaigns.

When a new drug or a vaccine is released onto the market, the only safety concerns reported will be those arising during a clinical trial, which typically has limits due to the small patient numbers, is conducted over a limited time period and has a long list of patient exclusion criteria. It is, therefore, essential to capture AEs that occur after the trial in a real-world setting. Pharmacovigilance systems have been set up to capture this information, including the Eudravigilance reporting system in Europe and the CDC or FDA VAERS in the United States. It is mandatory for manufacturers to report any AEs, but voluntary for some health care professionals and the public. Some patients may not know how to report AEs or know about the systems in place for reporting AEs; therefore, social media is an important data source that can be used to harness social reaction and, with the use of LM to observe AEs, as in this analysis. In addition, social media data can provide information on AEs in real time without any filter or having to wait for reporting through standard AE systems, which can take at least 2 months. This is particularly important in situations such as the COVID-19 pandemic when new vaccines released onto the market may not have completed lengthy trials, which is when many AEs are discovered. In this analysis, the data were collected soon after publication, that is, collated every week automatically and the use of the MedDRA terms allowed the identification of AEs using the same terms as traditional VAERS and Eudravigilance reporting systems.

There can be bias in pharmacovigilance systems, as not all patients are aware of spontaneous pharmacovigilance reporting. However, social media is widely accessible, and patients discuss AEs, especially during a pandemic. This provides the opportunity to detect AEs from social media to augment the bias in spontaneous reporting. However, social media is not structured to capture AEs the way spontaneous reporting systems are and even with context-aware LMs, false positives and false negatives can occur. Previous analyses of social media have been undertaken for AEs relating to Zika, Ebola, and dengue viruses. There is a suggestion, however, that illnesses less prevalent in the news may be better for prediction as there is less influence and bias through media outlets [35]. Although, some rare illnesses with a smaller population would need sufficient social media comments for meaningful analysis. Studies using social media listening have tended to focus on vaccine hesitancy and sentiment [14-22]. The work presented here using the Soteria web app has some similarities to previous studies, especially the work from Portelli et al [22], which, as our analysis, used continuous data collection and processing, global data collection, and used a transformer-pretrained LM for AE detection. However, our Soteria web app differs in that it includes a multitude of social media listening, including patient blogs and forums beyond X, it also uses AE coding using ICH standards (MedDRA) rather than focusing on sentiment. AAs well, it does not focus only on English but uses translation models to be able to include mentions from 190 countries in 61 different languages. Finally, the generation of trends using multiple dimensions (vaccine brand, mechanism, country, different hierarchy levels MedDRA, and special populations: pediatrics or pregnant women) and combinations of these dimensions (eg, AEs into a pediatric group who received Pfizer-BioNTech vaccine) can be undertaken via the graphical user interface using visual analytics. For example, the top serious AEs reported via VAERS for children aged 5-11 years who received the Pfizer-BioNTech COVID-19 vaccine, according to a published study [32], were incorrect dose, vomiting, fever, and headache, and in a parallel study for children aged 12-17 years were dizziness, syncope, nausea, headache, and fever [33]. These AEs align with the AEs observed using the Soteria web app. In addition, preliminary findings of mRNA COVID-19 vaccine safety in pregnant women have shown that the most frequently reported pregnancy-related AE was spontaneous abortion, again aligning with the AE observed in this analysis [34].

It is important to note that these AEs are not necessarily safety signals, as safety signals have a very specific definition: “information on a new or known AE that may be caused by a medicine and requires further investigation” (EMA) [36]. In fact, 1 study showed that the current methods of signal detection using social media did not perform well and could not be used to replace or integrate with the current pharmacovigilance activities [5]. However, these AEs observed from social media can potentially be of importance to adjust signal detection, assessment strategies, and communication with external stakeholders. For example, AEs observed from social media could augment and optimize existing signal detection processes in place, or even become the focus of signal assessment that traditionally uses data sources such as electronic health records. AEs observed via the Soteria app have the potential to inform the companies earlier than those of traditional postmarketing AE reporting systems, therefore, allowing early and timely alerts of rare AEs.

### Limitations

This study has limitations. The chatting habits are different between countries, for example, there was a higher availability of chats from the United States possibly leading to country bias. In addition, the frequency of chats may be affected by the media coverage within that country. As well, although social media and X reposts and reshares are only counted once, the same person may post the same AE several times, leading to over-reporting (although this can also occur within the VAERS system). Also, there may be false negatives due to incorrect translations (non-English sentences written using the standard English alphabet were removed as we were aware of poor performance with machine translation models at the time). Finally, these posts are not true diagnoses, and the people providing the chat may not experience the events or be aware of their medical diagnosis.

While BERT was the only available LM at the time of this work, this entire pipeline can be redone with novel LMs available today. Similarly, benchmark datasets for AE detection in social media are now available that can be used to measure the performance of the model. Further external validation of the model using these benchmark datasets could enhance the reliability of the model’s performance claims.

Future studies could include an artificial intelligence–based signal detection (instead of AE detection) with validation using more traditional methods and more commonly used data sources, such as VAERS, claims, and electronic medical records databases. Similar tools could be developed to monitor the safe use of vaccines other than COVID-19 vaccines or drugs.

Of note, as of November 23, 2022, X has not enforced their COVID-19 misinformation policy. A comparison of data before and after this date could be of interest, for example, to determine if the removal of this policy influenced vaccine AEs discussed within X.

### Conclusions

The application of LM to monitor web and social media data provides opportunities to observe AEs associated with COVID-19 vaccines faster compared to the traditional spontaneous reporting systems, which have a longer lag time between the occurrence of AEs and the availability of data. This gives the potential to enhance postmarketing surveillance. While AEs are not necessarily signals that require further analyses to confirm, they could help to adjust signal detection strategies by refocusing signal assessment on observed AEs and help to improve communication with external stakeholders, contributing to increased confidence in vaccines’ monitoring and safety. While “chatting” regarding AEs following COVID-19 vaccination is decreasing in social media, our LM-based AE detection model can be applied to other vaccines and medicines.

## Appendix

Multimedia Appendix 1 Languages available via Synthesio Ltd API and translated by Amazon Web Services translation or Helsinki-NLP model.

Multimedia Appendix 2 Vaccines monitored in Soteria app as of April 2022.

Multimedia Appendix 3 MedDRA lexical expansion.

Multimedia Appendix 4 COVID-19 vaccine related keywords used to query web content and social media (as of April 2022).

Multimedia Appendix 5 Word cloud by distribution of number of mentions with an AE (detected as MedDRA PT) between November 2020 and April 2022 by the vaccine platform. AE: adverse event; MedDRA: Medical Dictionary for Regulatory Activities; PT: preferred term.

Multimedia Appendix 6 Word cloud by distribution of number of mentions with an AE (detected as MedDRA PT) between November 2020 and April 2022 by vaccine brand. AE: adverse event; MedDRA: Medical Dictionary for Regulatory Activities; PT: preferred term.

Multimedia Appendix 7 Word cloud by cumulative count; selection criteria: PT, company. Extraction October 22, 2022. (A) Pfizer-BioNTech vaccine, (B) Moderna vaccine, (C) AstraZeneca vaccine, and (D) Janssen vaccine. PT: preferred term.

Multimedia Appendix 8 Word cloud by cumulative count. Selection criteria: PT, mRNA vaccines. Extraction April 2022. (A) Pediatric population and (B) pregnant women. mRNA: messenger ribonucleic acid; PT: preferred term.

## Abbreviations

ADE: adverse drug events
AE: adverse event
API: application programming interface
CDC: Centers for Disease Control and Prevention
EMA: European Medicines Agency
FDA: US Food and Drug Administration
GDPR: General Data Protection Regulation
HLGT: high-level group term
HLT: high-level term
ICH: International Council for Harmonization
LM: language model
MedDRA: Medical Dictionary for Regulatory Activities
mRNA: messenger ribonucleic acid
NLP: natural language processing
PIPL: Personal Information Protection Law
PsyTAR: psychiatric treatment adverse reactions
PT: preferred term
SOC: system organ class
UMLS: Unified Medical Language System
VAERS: Vaccine Adverse Event Reporting System
WHO: World Health Organization

## Data Availability

The datasets generated and analyzed during this study are available from the corresponding author on reasonable request.
