# Supplementary material for: Application of a Language Model Tool for COVID-19 Vaccine Adverse Event Monitoring Using Web and Social Media Content: Algorithm Development and Validation Study
Source: JMIR Infodemiology. 2024 Dec 20;4:e53424. doi: 10.2196/53424 (PMC11699502; doi:10.2196/53424)
Supplement: Multimedia Appendix 1 [file infodemiology_v4i1e53424_app1.docx]

| **Amazon Translate** | | **Amazon Translate or Helsinki-NLP** | | | **Helsinki-NLP** | **Not translated** | | |
| --- | --- | --- | --- | --- | --- | --- | --- | --- |
| Bosnian | Norwegian | Afrikaans | Estonian | Macedonian | Basque | Assamese | Javanese | Pushto |
| Traditional Chinese | Persian / Farsi | Albanian | Finnish | Maltese | Galician | Belarusian | Kinyarwanda | Romansh |
| Croatian | Portuguese | Arabic | French | Polish | Haitian | Breton | Kirghiz | Sankrit |
| Gujarati | Romanian | Armenian | Georgian | Russian | Irish | Burmese | Kurdish | Scots |
| Hebrew | Serbian | Azerbaijani | German | Slovak | Malagasy | Cebuano | Lao | Tajik |
| Kazakh | Sinhala | Bengali | Hindi | Spanish | Marathi | Central Khmer | Latin | Volapuk |
| Lithuanian | Slovenian | Bulgarian | Hungarian | Swedish | Panjabi | Simplified Chinese | Luxembourgish | Zulu |
| Malay | Somali | Catalan | Icelandic | Tagalog / Filipino | Waray | Corsican | Nepali (macrolanguage) |  |
| Malayalam | Swahili | Chinese | Indonesian | Thai | Welsh | Gaelic | Norwegian |  |
| Modern Greek | Tamil | Czech | Italian | Turkish |  | Interlingua | Occitan |  |
| Mongolian | Telugu | Danish | Japanese | Ukrainian |  | Interlingue | Oriya (macrolanguage) |  |
|  |  | Dutch | Korean | Urdu |  |  |  |  |
|  |  | English | Latvian | Vietnamese |  |  |  |  |

AWS, Amazon Web Services; API, Application Programming Interface; NLP, natural language processing.
