# Supplementary material for: Application of a Language Model Tool for COVID-19 Vaccine Adverse Event Monitoring Using Web and Social Media Content: Algorithm Development and Validation Study
Source: JMIR Infodemiology. 2024 Dec 20;4:e53424. doi: 10.2196/53424 (PMC11699502; doi:10.2196/53424)
Supplement: Multimedia Appendix 2 [file infodemiology_v4i1e53424_app2.docx]

**Multimedia Appendix 2:** Vaccines monitored in Soteria app as of April 2022

| **Mechanism** | **Company** | **Brand** |
| --- | --- | --- |
| Adenovirus-vectored | AstraZeneca | AstraZeneca Vaccine |
|  | CanSino Biologics | Ad5-nCoV |
|  | Gamaleya | Sputnik V |
|  | Janssen | J&J vaccine |
|  | ReiThera | GRAd-COV2 |
| DNA | Entos Pharmaceuticals | Covigenix VAX-001 |
| Inactivated virus | Bharat Biotech | Covaxin |
|  | Research Institute for Biological Safety Problems | QazCovid |
|  | Sinopharm | BBIBP-CorV |
|  | SinoVac | CoronaVac |
|  | Valneva | Valneva |
| mRNA | Academy of Military Science (AMS)\|Walvax Biotechnology\|Suzhou Abogen Biosciences | ARCoV |
|  | Chulalongkorn University | ChulaCov19 |
|  | Curevac | Curevac vaccine |
|  | Moderna | mRNA-1273 Moderna |
|  | Pfizer-BioNTech | Pfizer vaccine |
|  | Sanofi | Sanofi-TranslateBio Vaccine |
| mRNA or Protein vaccine | Sanofi | Sanofi unspecified |
| Protein | BioCubaFarma | Soberana 1 |
|  | BioCubaFarma | Soberana 2 |
|  | Brilife | Brilife |
|  | Novavax | NVX-CoV2373 |
|  | Sanofi | Sanofi-GSK Vaccine |
|  | UPMC \| University of Pittsburgh School of Medicine | PittCoVacc |
|  | Vektor State Research Center of Virology and Biotechnology | EpiVacCorona |
| VLP | Medicago | Medicago |
| Brand not mentioned | Brand not mentioned | Brand not mentioned |

DNA, deoxyribonucleic acid; mRNA, messenger ribonucleic acid; VLP, virus-like particles
