# Supplementary material for: Application of a Language Model Tool for COVID-19 Vaccine Adverse Event Monitoring Using Web and Social Media Content: Algorithm Development and Validation Study
Source: JMIR Infodemiology. 2024 Dec 20;4:e53424. doi: 10.2196/53424 (PMC11699502; doi:10.2196/53424)
Supplement: Multimedia Appendix 3 [file infodemiology_v4i1e53424_app3.docx]

**
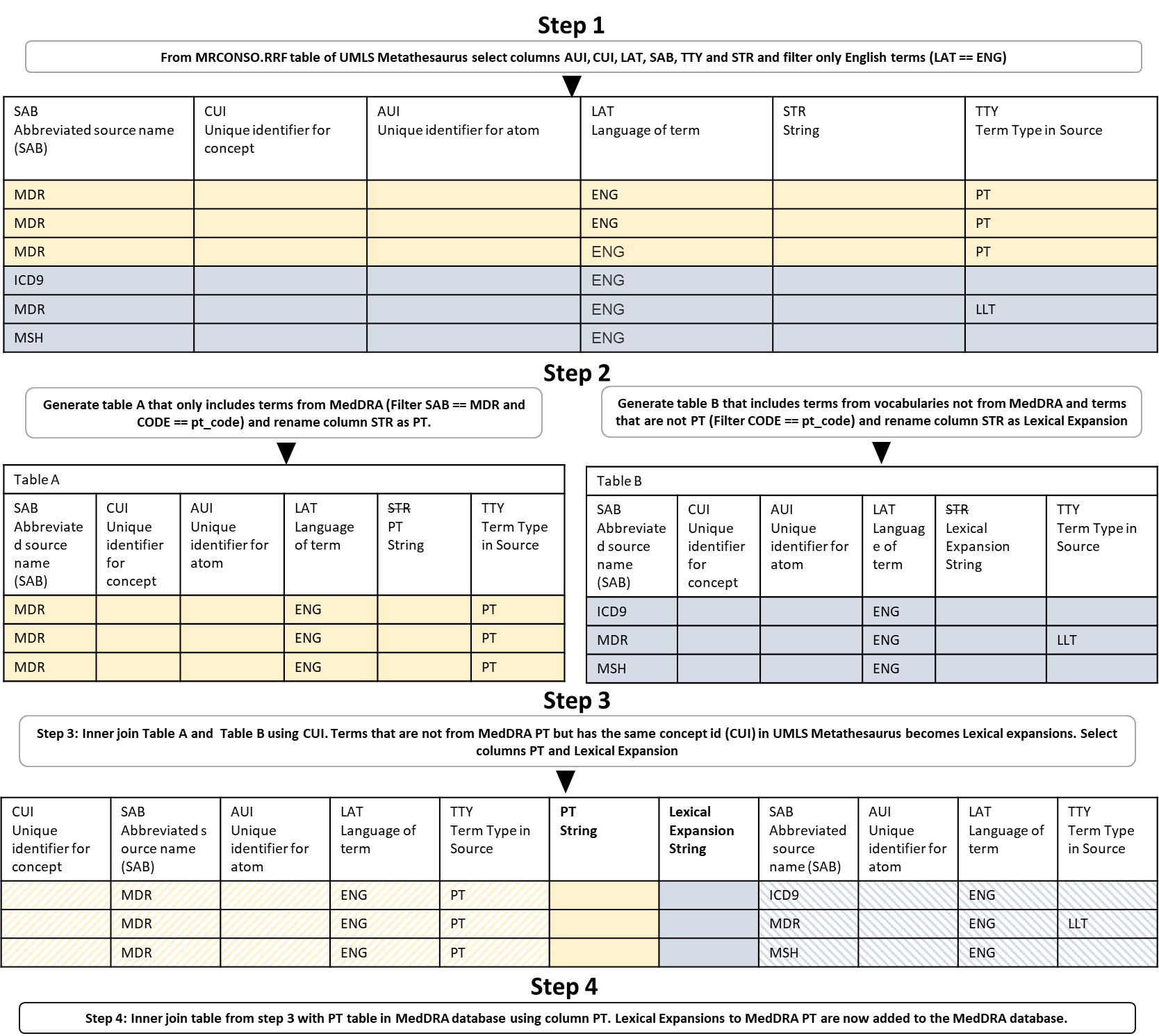
**

**E.g. In sentence, “I vomited food after COVID vaccine” is mapped to MedDRA PT “Vomiting” by the Soteria app using the** **MedDRA lexical expansion table as below.**

***I vomited food after COVID vaccine.***

**AE detection model**

***vomited food***

**Lexical matching to MedDRA**

**Lexical String**

**PT String**

**Vomiting food**

**Vomiting**

**Vomiting**

**Vomiting**

**Uncontrollable vomiting**

**Vomiting**

***vomited food***

***vomited food***

***vomited food***


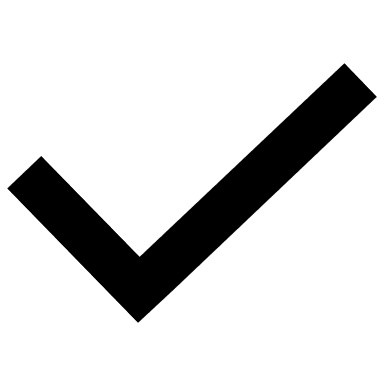

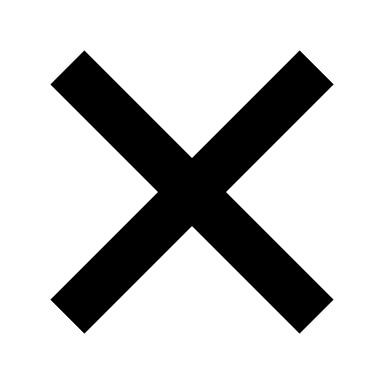

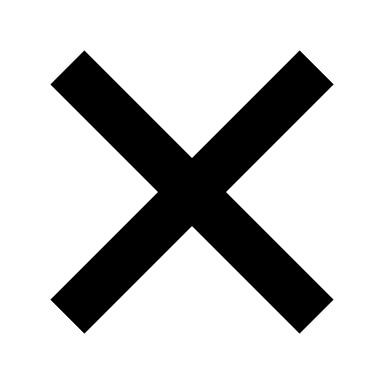


***Vomiting***

**MedDRA Lexical Expansion**
