# Supplementary figures and images for: Application of a Language Model Tool for COVID-19 Vaccine Adverse Event Monitoring Using Web and Social Media Content: Algorithm Development and Validation Study
Source: JMIR Infodemiology. 2024 Dec 20;4:e53424. doi: 10.2196/53424 (PMC11699502; doi:10.2196/53424)

**
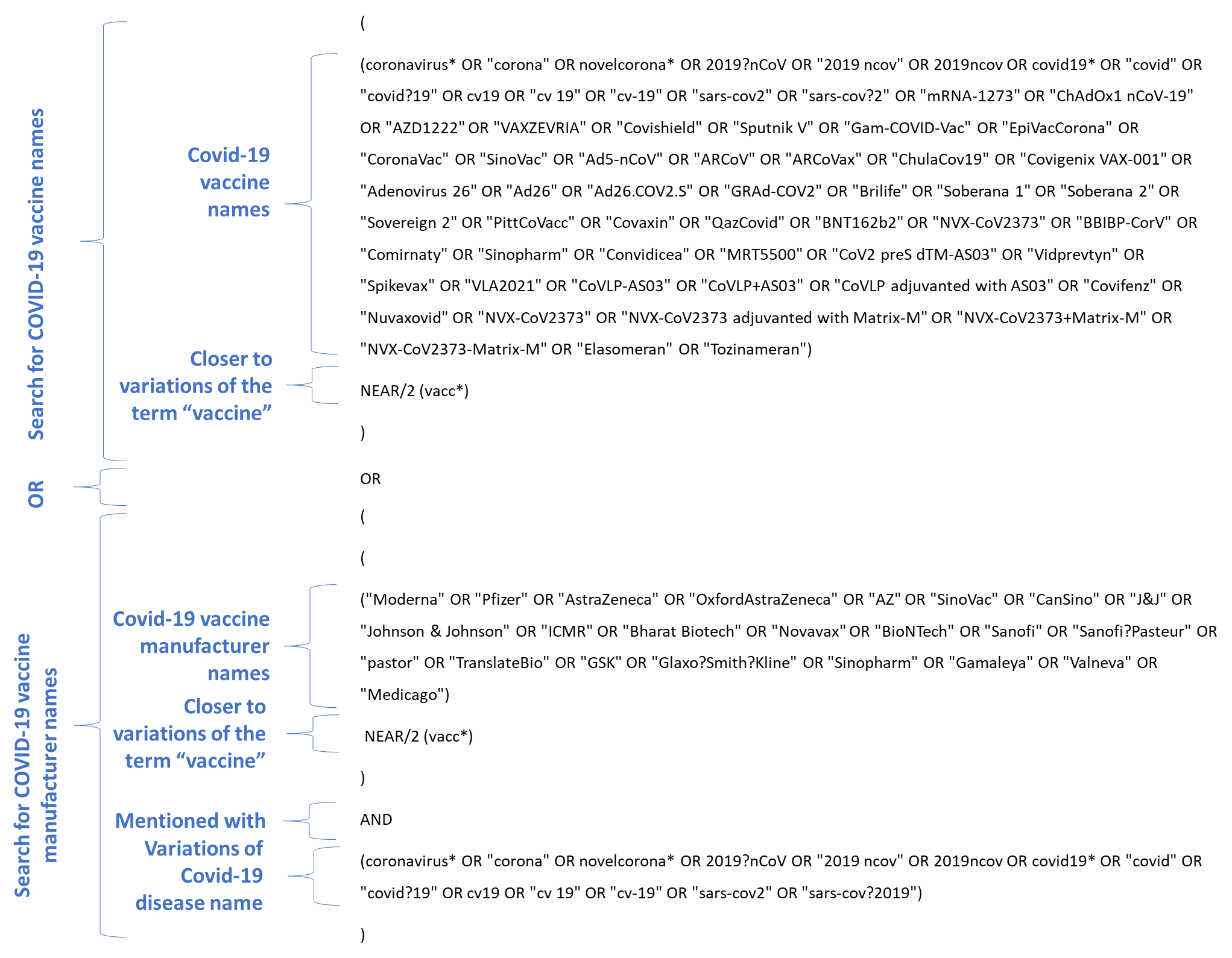
**

Supplement: Multimedia Appendix 4 [file infodemiology_v4i1e53424_app4.docx]

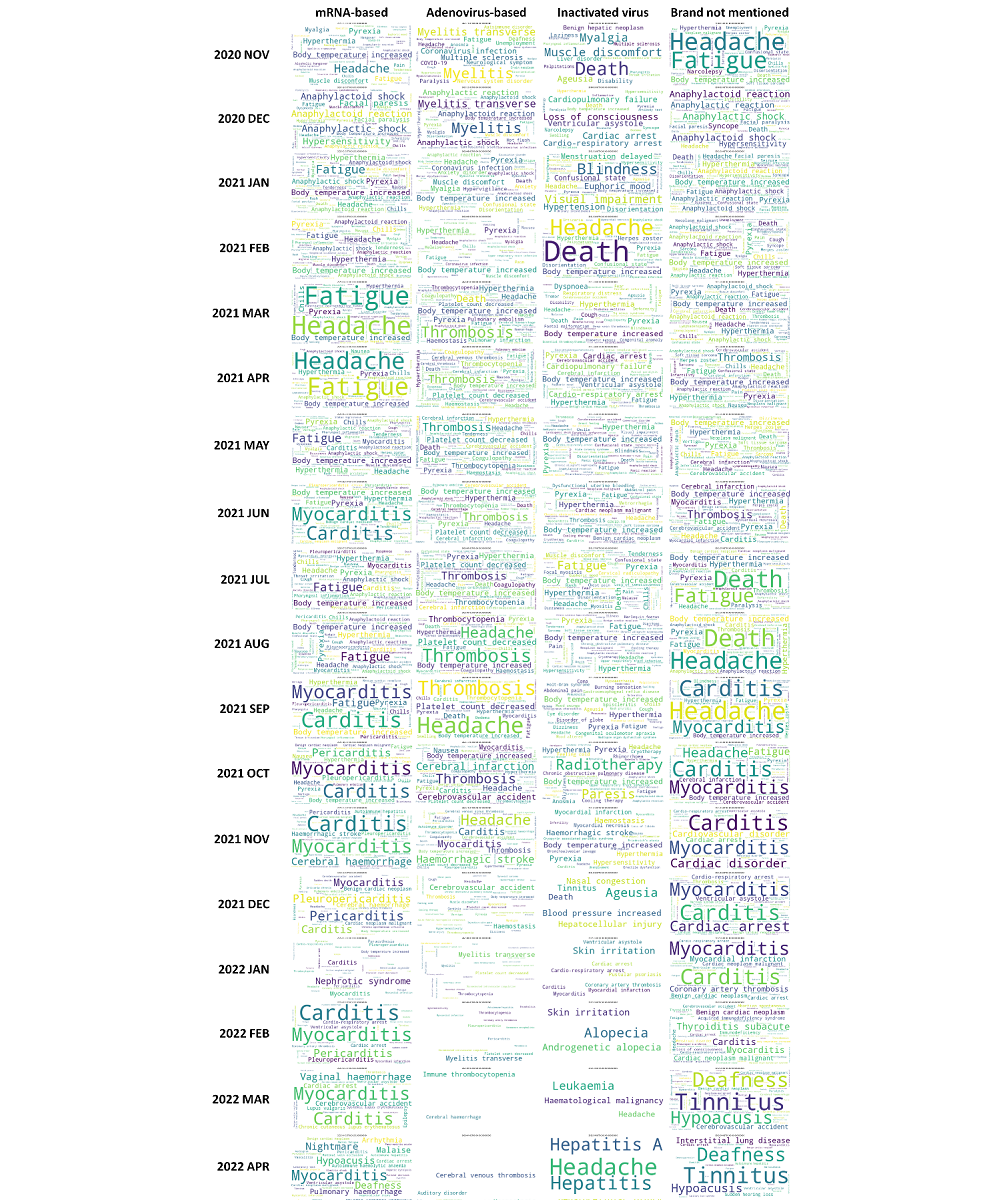

Supplement: Multimedia Appendix 5 [file infodemiology_v4i1e53424_app5.png]

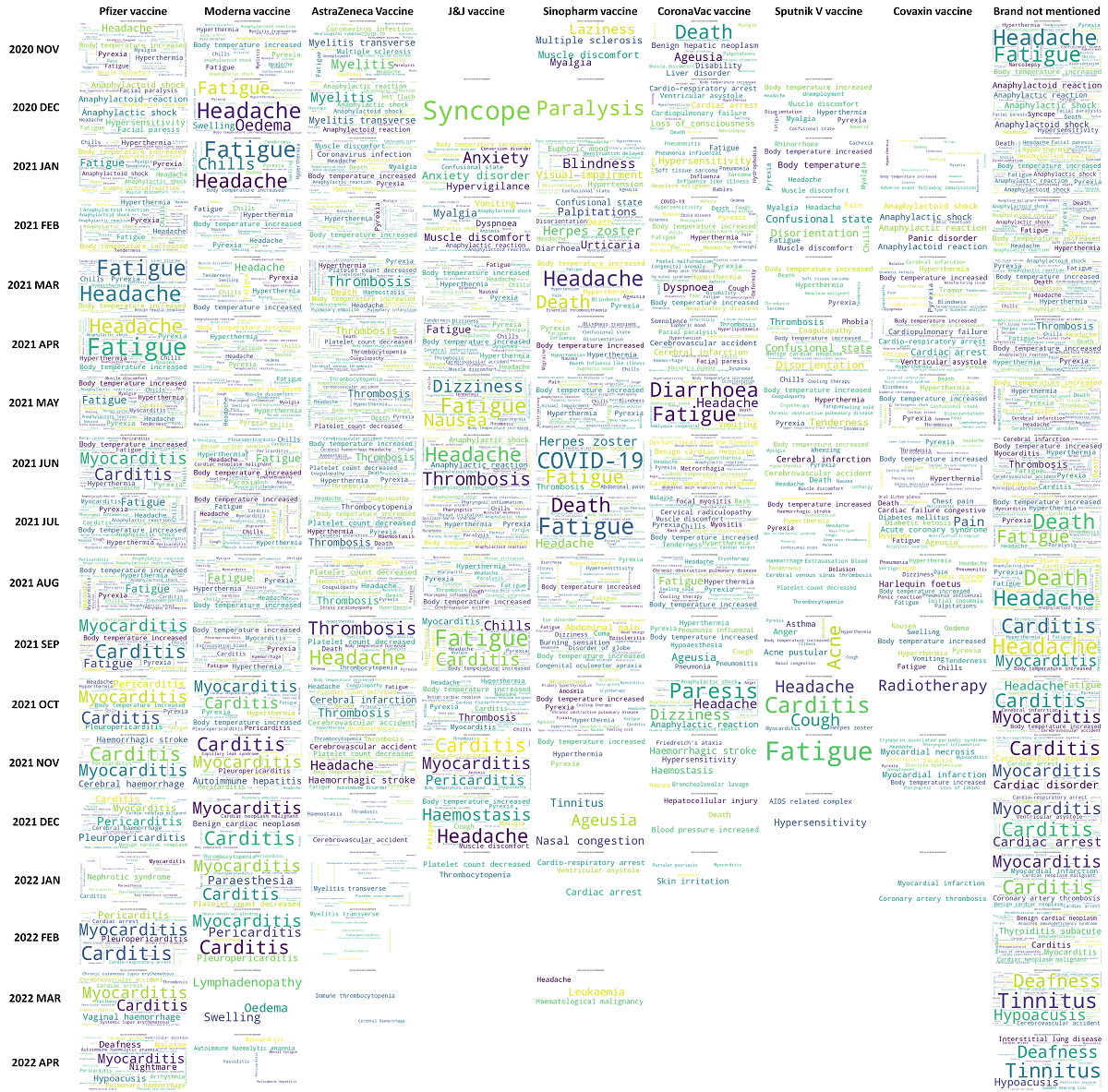

Supplement: Multimedia Appendix 6 [file infodemiology_v4i1e53424_app6.png]

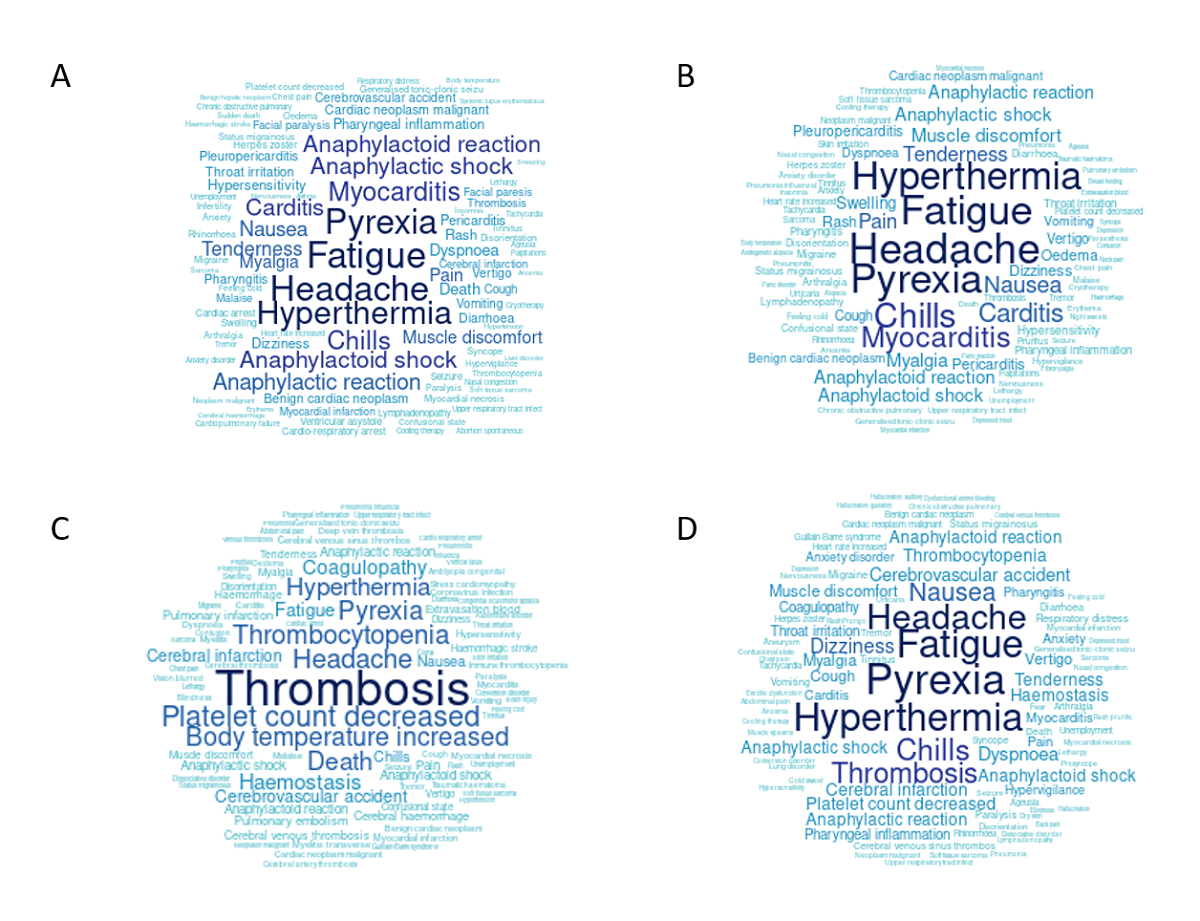

Supplement: Multimedia Appendix 7 [file infodemiology_v4i1e53424_app7.png]

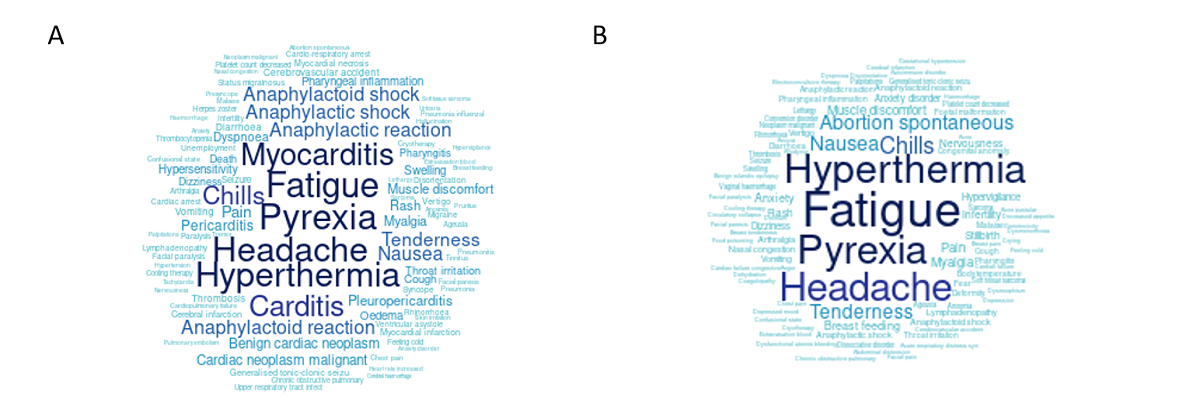

Supplement: Multimedia Appendix 8 [file infodemiology_v4i1e53424_app8.png]
